# Pressure ulcer prevention knowledge among Jordanian nurses: a cross- sectional study

**DOI:** 10.1186/1472-6955-13-6

**Published:** 2014-02-24

**Authors:** Jamal Qaddumi, Abdullah Khawaldeh

**Affiliations:** 1 Faculty of Medicine and Health sciences, An-Najah National University, P.O. Box 7, Nablus, Palestine; 2 Faculty of Nursing, Jarash University, Jarash, Jordan

**Keywords:** Pressure ulcer, Knowledge, Sources, Barriers, Nurses, Jordan

## Abstract

**Background:**

Pressure ulcer remains a significant problem in the healthcare system. In addition to the suffering it causes patients, it bears a growing financial burden. Although pressure ulcer prevention and care have improved in recent years, pressure ulcer still exists and occurs in both hospital and community settings. In Jordan, there are a handful of studies on pressure ulcer. This study aims to explore levels of knowledge and knowledge sources about pressure ulcer prevention, as well as barriers to implementing pressure ulcer prevention guidelines among Jordanian nurses.

**Methods:**

Using a cross-sectional study design and a self-administered questionnaire, data was collected from 194 baccalaureate and master’s level staff nurses working in eight Jordanian hospitals. From September to October of 2011, their knowledge levels about pressure ulcer prevention and the sources of this knowledge were assessed, along with the barriers which reduce successful pressure ulcer care and prevention.

ANOVA and *t*-test analysis were used to test the differences in nurses’ knowledge according to participants’ characteristics. Means, standard deviation, and frequencies were used to describe nurses’ knowledge levels, knowledge sources, and barriers to pressure ulcer prevention.

**Results:**

The majority (73%, n = 141) of nurses had inadequate knowledge about pressure ulcer prevention. The mean scores of the test for all participants was 10.84 out of 26 (SD = 2.3, range = 5–17), with the lowest score in themes related to PU etiology, preventive measures to reduce amount of pressure/shear, and risk assessment. In-service training was the second source of education on pressure ulcer, coming after university training. Shortage of staff and lack of time were the most frequently cited barriers to carrying out pressure ulcer risk assessment, documentation, and prevention.

**Conclusions:**

This study highlights concerns about Jordanian nurses’ knowledge of pressure ulcer prevention. The results of the current study showed inadequate knowledge among Jordanian nurses about pressure ulcer prevention based on National Pressure Ulcer Advisory Panel guidelines. Also, the low level of nurses’ pressure ulcer knowledge suggests poor dissemination of pressure ulcer knowledge in Jordan, a suggestion supported by the lack of relationship between years of experience and pressure ulcer knowledge.

## Background

Pressure ulcer (PU) still exist as a pervasive problem and occurs in both hospital and community settings, affecting all age groups, but mostly occurring among the elderly, the immobile, and those patients with severe acute illness or neurological deficits [1]. Pressure ulcer remains a significant health problem causing suffering for patients and a growing financial burden [2]. Pain and distress from PU are viewed as indications of poor PU prevention practice, since they restrict a patient’s lifestyle; PU prevention should be regarded as a priority in clinical and non-clinical areas, especially where patients are at high risk [3].

Nurses are often found to demonstrate poor adherence to the PU prevention guidelines [4-10]. The compliance of nurses to the guidelines was found to be influenced by several barriers [11,12]. A lack of knowledge is an apparent barrier for using the guidelines in clinical practice [7,8].

Limited application of knowledge is a common problem in clinical practice [12]. Nurses are not fully aware the importance of using up-to-date PU prevention protocols and may not have been exposed to current evidence-based practices; sometimes their practices can be influenced by intuition, experience, or habit [13]. Jordan O’Brien and Cowman [14] found a significant gap between nursing records of skin condition, and actual skin examination in relation to PU, which means that nurses were unable to identify the early signs of PU development. Hill [15] concluded that lack of knowledge and insufficient equipment are considered barriers that prevent healthcare providers from maintaining effective PU prevention and treatment.

Lack of knowledge and skills in PU prevention contributes significantly to the occurrence or worsening of PU; therefore, nurses require regular training and education in this area of practice [13]. Moreover, increased knowledge about PU prevention among nurses not only improves the quality of PU care but also reduces hospital stays, and the number of patients suffering from this condition [16]. Beeckman et al. [17] declared that adequate knowledge about PU prevention is important for deciding: (1) which patients should receive prevention, (2) which prevention should be applied, and (3) how prevention should be applied. Although PU education improves knowledge, studies have also shown that regular educational updates are needed to maintain and improve PU knowledge and practice standards [18]. Within Jordanian healthcare settings, there is a dearth of information relating to PU prevention. There are few studies on PU in Jordan, but one such study [19] found that the prevalence rate of PU in Jordan was 12% and emphasised the need to raise the awareness of nurses regarding PU.

### Objectives

The objective of this study was to explore the knowledge levels and sources of knowledge about PU prevention, as well as the barriers to implementing PU prevention guidelines among Jordanian nurses.

## Methods

### Study design

A cross-sectional survey was used; approval was obtained from the Institutional Review Board at the University of Jordan.

### Setting and sample

**Figure 1:**
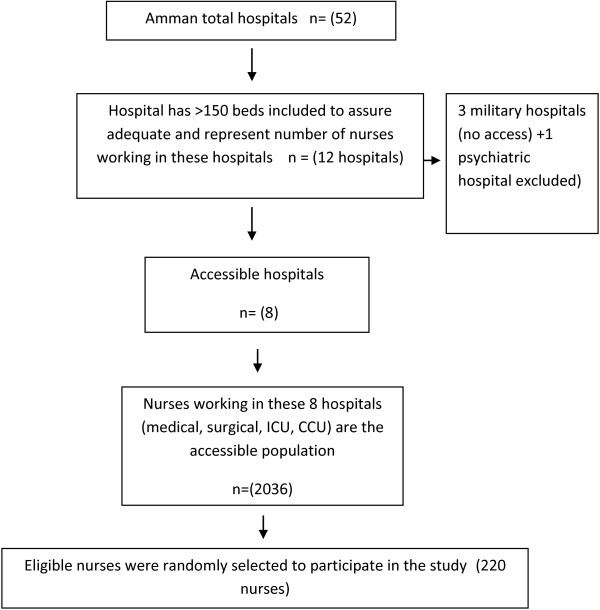
Study sampling and data collection points.

The data in this study pertain to nurses working in hospitals of the Amman Region of Jordan. Amman is the capital of Jordan and is located in the middle part of Jordan. It contains 52 hospitals (50% of the total hospitals in Jordan) with a total capacity of 6,305 beds according to the Jordan ministry of health (MOH) annual statistical report [20]. Only those hospitals having Medical, Orthopedic, Intensive Care, Burns, Surgical, or Coronary Care units and also having 150 beds or more were included to ensure exposure of participants to PU cases, and to represent adequately the nurses working in the region. Only eight hospitals were included in the study out of the 52 hospitals in Amman, as not all of them had 150 beds or more. The steps involved in selecting hospitals are illustrated in Figure 1.

The accessible population consisted of all eligible nurses who worked in the eight selected hospitals. The nursing staff working directly with adult patients at the eight selected hospitals comprised 2,153 baccalaureate and master’s level nurses.

A number of nurses were randomly selected (N = 220) by picking names from the staff lists at the eight selected hospitals. A random sample of participants was drawn from the population of nurses who met the eligibility criteria. The inclusion criteria was: (a) those male and female nurses who have acquired a bachelor’s or master’s degree in nursing, (b) who provide direct care nursing in their units (Medical, Orthopedic, Intensive Care, Burns, Surgical, and Coronary Care), and (c) who have at least one year of clinical experience.

### Study instrument

A questionnaire was developed consisting of a demographic characteristics part which included gender, age, years of clinical experience, level of current higher education, previous participation in PU research, sources of knowledge, and recent exposure to PU education.

The second part was the PU Knowledge Test Tool, English version, which was developed and validated by Beeckman et al. [17]. This test tool was used to examine the nurses’ knowledge level about PU by the sum of total correct answers. The test includes 26 multiple-choice questions reflecting six themes that express the most relevant aspects of PU. Questions and answers were based on evidence-based literature and conform to the 2009 international guidelines for PU prevention [21,22]. The instrument multiple-choice questions have 3 answer options, and the fourth was “I do not know the answer” option. The fourth answer was included to avoid the respondents guessing. Correct answer scored one point while incorrect one scored zero. The tool was validated for difficulty, discriminating index, and quality of the response alternatives [17]. The internal consistency reliability (Cronbach’s α) was 0.77 and the 1-week test-retest interclass correlation coefficient (stability) was 0.88. Content validity index (CVI) was 0.78–1.00. The item difficulty index of the questions ranged from 0.27 to 0.87, while values for item discrimination ranged from 0.29 to 0.65 [17].

The third part was a list of knowledge sources on pressure ulcer prevention (the sources from which nurses obtained their PU prevention knowledge). This was used to explore the common sources used by nurses to gain knowledge on PU prevention, and allowed respondents to choose more than one source.

The fourth part was a list of barriers to the implementation of PU prevention. It was used to measure barriers related to assessing, documenting, and carrying out PU prevention practices. This allowed respondents to rank the most important three barriers in each category. The third and fourth parts of the questionnaire were taken from an instrument developed and validated by Moore and Price [10].

### Data collection

An invitation with a full written description of the study was sent to the hospitals through the Jordanian Nurses and Midwifery Council (JNMC). The hospitals which agreed to participate were asked to send a list of their nurses, including those nurses who met the eligibility criteria and accepted participation. Before participation, all participants signed a written consent form after they had received an explanation about the research, the voluntary nature of their participation, and a guarantee of anonymity. These data collection points are illustrated in Figure 1. The questionnaire was administered in English because all nurses in Jordan are taught nursing in the English language.

### Data analysis

Statistical analyses were performed using the Statistical Package for Social Sciences (SPSS) version 17. A *p* value < 0.05 was considered statistically significant. Descriptive statistics were used to draw summary measures of central tendency and frequencies for demographic items. Nurses’ knowledge of PU prevention was assessed using the *t* test to compare the nurses’ knowledge scores between the two levels’ variables (gender, level of higher education, and previously involved in PU research), and the Analysis of Variance (ANOVA) test to compare the three or more levels’ variables (age, sources of education on PU, and years of clinical experience). Nurses’ knowledge of PU prevention was determined by calculating the total and mean scores. Since each theme of PU knowledge has different number of items, Z score used to compare the level of nurses’ PU knowledge between the six themes of PU knowledge. Nurses’ perceived barriers to the implementation of PU prevention were assessed by calculating the frequency and percentage of the three most important perceived barriers as ranked by nurses.

## Results

### Participants’ characteristics

The sample consisted of 194 eligible nurses who provide direct bedside care for patients. Table 1 shows demographic details of the sample. The majority of participants were males (n = 114, 58.8%). Age ranged from 22 to 40 years, with a mean age of 27.3 years (SD = 3.47, range = 22–40). The majority had a bachelor’s degree (88.1%, n = 171), while other nurses had master’s degrees (11.9%, n = 23), and 36.6% (n = 69) of the nurses reported that they had not received training or education about PU. Most of the participants (93.8%, n = 182) had clinical nursing experience of between one to ten years, and 45.9% (n = 89) had one to four years, while 47.9% (n = 91) had five to ten years of clinical nursing experience. The majority of participants (84.0%, n = 163) had not previously participated in PU research.

**Table 1:** Demographic details of the sample (N = 194)

**Nurses’ characteristics**	**%**	**n**
Gender		
Male	114	58.8
Female	80	41.2
Age		
20-25 years	67	34.5
26-30 years	99	51.2
31-35 years	25	12.8
36-40 years	3	1.5
Current higher degree		
Bachelor	171	86.0
Master	23	14.0
Nursing clinical experience		
< 2 year	10	5.1
2-4 years	89	45.8
5-10	93	47.9%
11-15	1	0.5
16-20	1	0.5
Source of PU education		
University	111	57.2
In-service	45	32.1
Conference	6	3.0
Product pro	11	5.6
other	4	2.0
PU research		
Yes	23	35.6
No	125	64.4
Last attend training on PU		
< 1 year	78	82.9
1-2 year	14	7.2
> 2 year	28	14.4
Never	69	35.5

PU, pressure ulcer.

The statistical analysis of the participants’ demographics revealed that there was no significant relationship between nurses’ knowledge of PU prevention and their age, clinical nursing experience, current higher education, PU research participation, and last attendance at PU training. In contrast, gender had a significant relationship with nurses’ knowledge of PU prevention (see Table 2).

**Table 2:** Differences in PU prevention knowledge and participants’ characteristics (N = 194)

**Nurses' characteristics**	**n**	**M**	**SD**	**t-test**	**ANOVA**	**P-value**
Gender						
Male	114	11.4	2.2	2.33		0.021*
Female	80	10.5	2.1			
Age						
20-25 years	67	11.4	2.0		1.12	0.13
26-30 years	99	10.7	2.1			
31-35 years	25	11.6	2.6			
36-40 years	3	12.0	1.4			
Current higher degree						
Bachelor	167	10.6	2.0	-1.25		0.21
Master	23	11.4	2.7			
Nursing clinical experience						
	< 2 year	10	11.4	2.0		0.41	0.66
	2-4 years	89	11.4	1.7			
	5-10	93	10.7	2.6			
	11-15	1	10.0				
	16-20	1	11.0				
Source of PU education							
University	111	11.4	2.2	1.0		0.42	
In-service	45	11.0	1.8				
Conference	6	10.2	2.6				
Product	11	10.3	1.5				
other	4	11.0	0.0				
PU research							
Yes	23	10.8	2.2	1.79		0.075	
No	125	9.9	2.1				
Last attend training on PU							
< 1 year	78	10.7	2.3		1.42	0.23	
1-2 year	14	11.1	2.5				
> 2 year	28	11.4	1.7				
Never	69	11.4	2.1				

*Significant at the 0.05 level (2-tailed).

**Significant at the 0.01 level (2-tailed).

PU, pressure ulcer.

### Nurses’ knowledge of PU prevention

**Figure 2:**
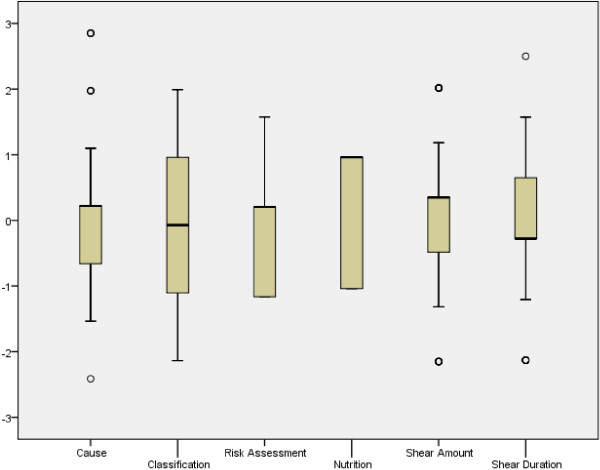
**Distribution of mean Z score of the six themes of PU knowledge test.** **Significant at the 0.01 level (2-tailed). PU = Pressure Ulcer.

The possible score of the PU Knowledge Test ranged from 0 to 26. The results of the PU Knowledge Test were calculated for the nurses who participated in the study (N = 194). The mean score for all participants was 10.84 out of 26 (SD = 2.3, range = 5–17). The passing score was 13 (50%): 142 (73%) of participants did not pass the test, while 27% (n = 52) did pass the test (with a score range of 13 to 17). The PU Knowledge Test showed low level in PU knowledge among nurses with the lowest score in themes related to PU etiology, preventive measures to reduce amount of pressure/shear, and risk assessment. Other themes of PU Knowledge Test scores as classification, nutrition, and preventive measures to reduce the duration of pressure/shear) had higher scores but still low. Twenty five percent of nurses did pass the Z score (.20) in PU risk assessment, Z score (.22) in PU etiology (n = 100), Z score (.35) in PU preventive measures to reduce amount of pressure/shear, Z score (.65) in PU preventive measures to reduce duration of pressure/shear, and Z score (.96) in both the PU preventive classification and PU nutrition (See Figure 2).

### Nurses’ sources of knowledge on PU prevention

The results presented in Table 3 revealed that most of the participants 51% (n = 99) did not receive any type of education on PU after graduation from university. In-service training is the second source of education on PU (32%, n = 63), coming after university at 51% (n = 99).

**Table 3:** Knowledge Sources on PU (N = 194)

**Sources of pressure ulcer prevention education**	**Number (%)**
Total number of respondents	194 (100)
University	99 (51.1)
In-service training	50 (25.9)
In-service training plus degree	13 (6.7)
Product promotion	11 (5.9)
Conference	7 (3.7)
Other	4 (2.2)

*PU*, pressure ulcer.

### Nurses’ perceived barriers to implementing PU prevention

Barriers to implementing PU prevention were measured using a list of barriers related to assessing, documenting, and carrying out PU prevention practices. The most commonly cited possibilities were lack of time (34.1%, n = 46), shortage of staff (24.4%, n = 33), the patient’s condition (17.8%, n = 24), and lack of resources or equipment (19.3%, n = 26).

Potential barriers to carrying out PU risk assessment, PU documentation, and PU prevention are presented in Table 4. Shortage of staff and lack of time were the most frequently cited barriers to carrying out PU risk assessment (48.1%, n = 93), documentation (56.3%, n = 109), and prevention (39.2%, n = 76). Barriers related to patients were the third most frequently cited after staff and time: for instance, the patient may be too ill to be assessed or may be uncooperative (14.8%, n = 20), making assessment difficult. Lack of training and lack of aides (15.6%, n = 21) were also perceived as important barriers. However, lack of knowledge (9.6%, n = 13) was mentioned as at least as important a barrier for carrying out PU risk assessment, documentation, and prevention.

**Table 4:** Barriers to carrying out PU* risk assessment, PU documentation, and PU prevention (N = 194)

**Barriers to carrying out PU**	**Risk assessment number (%)**	**Documentation number (%)**	**Prevention number (%)**
Total number of respondents	194 (100)	194 (100)	194 (100)
Lack of time	32 (23.7)	46 (34.1)	33 (24.4)
Unstable patient	17 (12.6)	16 (11.9)	24 (17.8)
Lack of training, resources, equipment, guidelines	26 (19.3)	0 (0)	22 (16.3)
Short staffed	33 (24.4)	30 (22.2)	20 (14.8)
Lack of knowledge	13 (9.6)	14 (10.4)	19 (14.1)
Lack of aids	0 (0)	15 (11.1)	14 (10.4)
Unable to assess	9 (6.7)	0 (0)	13 (9.6)
Problem with assessment tool	9 (6.7)	21 (15.6)	0 (0)
Forget	0 (0)	13 (9.6)	0 (0)
Lack of equipment	18 (13.3)	10 (7.4)	0 (0)
Patient un-cooperative/too ill	20 (14.8)	0 (0)	0 (0)

*PU, pressure ulcer.

## Discussion

### Nurses’ knowledge of PU prevention

Apparently, knowledge about PU prevention is poor and not very well known among nurses [4,23]. Pressure ulcer prevention rarely seems to be based on scientific evidence, but rather on expert opinion and tradition [9].

A 50% cutoff point (answering 13 out of 26 items on the PU knowledge test correctly) was used to identify nurses’ PU knowledge, which was considered low compared to similar results in the relevant literature [4]. Although other studies had higher cutoff, only 27% (n = 52) of the nurses passed the test. A higher cutoff point may pose serious questions about nurses’ PU knowledge.

Although the results of the current study were similar to those of Pieper and Mott [23], Panagiotopoulou and Kerr [5], Abou El Enein and Zaghloul [24], and Beeckman et al. [17], different methods, a different knowledge test, and different evaluation criteria were used. For example, Pieper and Mott [23] examined nurses’ knowledge of PU prevention and staging using a PU knowledge test and found that nurses had poor knowledge about PU prevention and staging. In more recent studies [17,24], results revealed that nurses’ knowledge was poor and inadequate. While Abou El Enein and Zaghloul [24] found that nurses’ knowledge about PU prevention was below the cutoff point they established (70%), Beeckman et al. [17] used a lower cutoff point (60%) and reported similar results. These studies suggested that nurses’ knowledge about prevention of PU must be increased and guidelines should be implemented in clinical practice. Different outcomes came from Sinclair et al. [18] and Gunningberg [12], who assessed nurses’ knowledge on PU care and reported that nurses’ knowledge was moderate.

Numerous factors could be contributing to the lack of nurses’ knowledge revealed in the current study. One is related to educational opportunities, including the availability, timing, and cost of education, as well as the associated staffing issues. Furthermore, staff turnover has increased in the last five years [25], making it difficult for a facility to maintain necessary PU educational programmes and to maintain a staff educational base related to up-to-date PU prevention. Hayajneh et al. [25] considered the turnover of Jordanian registered nurses in hospitals a significant problem that requires effective strategies to resolve.

An additional aspect in PU prevention is the Risk Assessment Scale (RAS). Risk assessment tools along with advanced PU prevention measures are not available in most Jordanian hospitals. The fact that nurses were not well oriented with such advanced measures and using the PU RAS could also explain their lack of knowledge about PU prevention. This lack of knowledge could lead to less than optimal care, especially if nurses use and practice outdated methods and/or inconsistent therapies.

Moreover, a lack of both tissue viability nurse specialists in Jordan and national PU guidelines may impact PU prevention in Jordan through inadequate knowledge and an absence of updated, evidence-based practice in this area of specialisation.

### Nurses’ knowledge of PU prevention and their demographics

The current study showed few differences in knowledge scores with regard to nursing education, years in practice, PU training, age, or source of knowledge on PU prevention, which confirms the results of Pieper and Mott [23] and Hulsenboom, Bours, and Halfens [26]. Pieper and Mott [23] evaluated the knowledge of PU prevention and staging among nurses. Their results revealed that nurses’ knowledge had no relations with nurses’ education, age, or years of work experience. This may be limited to their study sample. The sample did not include non-professional staff who may be less likely to attend continuing education or who may have greater problems with literacy and providing PU care in clinical practice. Hulsenboom, Bours, and Halfens [26] found that demographic variables, including the age and experience of nurses, had no significant influence on PU prevention interventions.

The present study is inconsistent with the findings of Choa, Parkb, and Chunge [27], who analysed nurses’ characteristics in relation to PU prevention and found that more PU prevention was documented by those who were younger, less experienced, and more educated.

In the current study, the non-significant influence of age, previous participation in PU research, and level of education on nurses’ PU knowledge could be explained by unequal representation between the levels in these variables. The sample of nurses included only 11.9% (n = 23) with a master’s degree compared to 88.1% (n = 167) with a bachelor’s degree. The age of the majority of nurses was between 25 and 30 years. The turnover rate of registered nurses in Jordan [25] in addition to the inclusion of only those nurses who provide bedside care could have contributed to the young age of the sample participants. An additional impact on inadequate knowledge of PU prevention among nurses may have arisen from the unequal proportions in these variables, and might have contributed to the non-significant results.

### Barriers to implementing PU prevention

The dissemination of knowledge about PU prevention among nurses was found to be influenced by barriers related to the use of guidelines, lack of staff, and lack of time. Similar results were found by Moore and Price [10], who pointed out a gap between theory and practice despite nurses’ positive attitudes toward PU prevention due to barriers such as a lack of staff and time. Compared to Halfens and Eggink [4] and Abou El Enein and Zaghloul [24], the current study concludes that despite the increased attention and new developments in the area of PU care, knowledge of PU prevention is still low and has not significantly increased.

In this study, lack of time and shortage of staff were first and most commonly cited as nurses’ perceived barriers to carrying out PU prevention, whilst lack of training and education was ranked second. These findings were supported by the result of Jordan O’Brien and Cowman [14], who found that a lack of time and staff was the main barrier to the completion of nursing documentation of PU care plans. Moreover, the ward rounds reduced the time for documenting the delivery of care [14].

Pressure ulcer prevention is an interdisciplinary problem. Thus, it needs multidisciplinary efforts and team work to contribute to successful care. An additional problem is created by staff shortages, which result in work overloads for staff at the clinical level. Certain aspects of PU prevention, such as repositioning, are difficult to carry out unaided. If staff shortages continue, with the stress caused during the busy and overloaded clinical shifts, it will be no surprise if PU prevention becomes less of a priority.

This study highlights concerns about Jordanian nurses’ knowledge of PU prevention. The results of the current study showed inadequate knowledge among Jordanian nurses of PU prevention based on National Pressure Ulcer Advisory Panel guidelines. Also, these findings suggest poor dissemination of PU knowledge in Jordan, which the lack of relationship between years of nursing experience and PU knowledge seems to substantiate.

### Limitations

Our study has some limitations, such as the sample selection being limited to Amman hospitals and the use of a self-administered questionnaire. However, the researcher believes that the results of this study can be applied to all nurses working in the Jordan healthcare system since nurses in Amman hospitals are similar to those of other Jordan regions in that there are no great regional differences in the type of education that nurses receive.

## Conclusions

In the current study, the majority of the nurses did not have sufficient knowledge to demonstrate competency in PU prevention. In fact, too few nurses achieved the minimum score (50%; 13 out of 26 correct answers) required to pass the PU knowledge test. The findings demonstrated that nurses are not equipped with enough education to predict and prevent PU appropriately. This supports the need to implement a PU educational programme in Jordanian healthcare settings to improve patients’ outcomes. In conclusion, Jordanian nurses’ knowledge about PU prevention was inadequate. Furthermore, adequate dissemination of PU prevention guidelines seems to be a prerequisite to improving the quality of PU prevention. Improving practice requires a multi-faceted approach to ensure adequate support to make changes based on patients’ outcomes. Further research on PU prevention in healthcare settings is needed.

## Competing interests

The authors declare that they have no competing interests.

## Authors’ contributions

JQ has made substantial contributions to the conception and design of the study, the acquisition of data, the analysis and interpretation of the data, and the drafting of the manuscript. AK has also made substantial contributions to data collection and interpretation, and to drafting the manuscript. Both authors have given final approval of the version to be published.

## Pre-publication history

The pre-publication history for this paper can be accessed here:

http://www.biomedcentral.com/1472-6955/13/6/prepub

## References

[B1] European Pressure Ulcer Advisory Panel, National Pressure Ulcer Advisory Panel Prevention and Treatment of Pressure Ulcers: Quick Reference Guide 2009 Washington, DC: National Pressure Ulcer Advisory Panel http://www.epuap.org/guidelines/Final_Quick_Treatment.pdf

[B2] Spilsbury K Nelson A Iglesias C Nixon J Mason S Pressure ulcers and their treatment and effects on quality of life: hospital inpatient perspectives J Adv Nurs 2007 57 5 494 504 10.1111/j.1365-2648.2006.04140.x 17284276

[B3] Hopkins A Dealey C Bale S Defloor T Worboys F Patient stories of living with a pressure ulcer J Adv Nurs 2006 56 4 345 353 10.1111/j.1365-2648.2006.04007.x 17042814

[B4] Halfens RJG Eggink MC Knowledge, beliefs and use of nursing methods in preventing pressure sores in Dutch hospitals Int J Nurs Stud 1995 32 16 26 10.1016/0020-7489(94)00032-F 7730002

[B5] Panagiotopoulou K Kerr SM Pressure area care: an exploration of Greek nurses’ knowledge and practice J Adv Nurs 2002 40 3 285 296 10.1046/j.1365-2648.2002.02370.x 12383180

[B6] Meesterberends E Halfens R Lohrmann C de Wit R Pressure ulcer guideline development and dissemination in Europe J Clin Nurs 2010 19 11–12 1495 1503 20579194 10.1111/j.1365-2702.2010.03229.x

[B7] Ajzen I Madden TJ Prediction of goal-directed behaviour: attitudes, intentions and perceived behavioural control J Exp Soc Psychol 1986 22 453 474 10.1016/0022-1031(86)90045-4

[B8] Buss IC Halfens RJG Huijer Abu-Saad H Kok G Pressure ulcer prevention in nursing homes: views and beliefs of enrolled nurses and other health care workers J Clin Nurs 2004 13 668 676 10.1111/j.1365-2702.2004.00953.x 15317506

[B9] Stephens F Bick D A national pilot to implement pressure ulcer guidelines: results of the baseline audit Br J Community Nurs 2002 7 Suppl 3 34 38 12514499 10.12968/bjcn.2002.7.sup3.10898

[B10] Moore Z Price P Nurses’ attitudes, behaviors and perceived barriers towards pressure ulcer prevention J Clin Nurs 2004 13 942 952 10.1111/j.1365-2702.2004.00972.x 15533100

[B11] Van Gaal BG Schoonhoven L Vloet LC Mintjes JA Borm GF Koopmans RT van Achterberg T The effect of the SAFE or SORRY? programme on patient safety knowledge of nurses in hospitals and nursing homes: a cluster randomized trial Int J Nurs Stud 2010 47 9 1117 1125 10.1016/j.ijnurstu.2010.02.001 20202633

[B12] Gunningberg L Are patients with or at risk of pressure ulcers allocated appropriate prevention measures? Int J Nurs Prac 2005 11 58 67 10.1111/j.1440-172X.2005.00503.x 15752320

[B13] Gunningberg L Pressure ulcer prevention: evaluation of an education programme for Swedish nurses J Wound Care 2004 13 3 85 89 15045800 10.12968/jowc.2004.13.3.26587

[B14] Jordan O’Brien JA Cowman S An exploration of nursing documentation of pressure ulcer care in an acute setting in Ireland J Wound Care 2011 20 5 197 205 21647065 10.12968/jowc.2011.20.5.197

[B15] Hill L Wound care nursing. The question of pressure Nurs Times 1992 88 12 76 82 1561145

[B16] Smith D Waugh S Research study: an assessment of registered nurses’ knowledge of pressure ulcers prevention and treatment Kansas Nurs 2009 84 1 3 5

[B17] Beeckman D Vanderwee K Demarre´ L Paquay L Van Hecke A Defloor T Pressure ulcer prevention: development and psychometric validation of a knowledge assessment instrument Int J Nurs Stud 2010 47 4 399 410 10.1016/j.ijnurstu.2009.08.010 19781701

[B18] Sinclair L Berwiczonek H Thurston N Butler S Bulloch G Ellery C Giesbrecht G Evaluation of an evidence-based education program for pressure ulcer prevention J Wound Ostomy Continence Nurs 2004 31 1 43 50 10.1097/00152192-200401000-00007 15128094

[B19] Tubaishat A Anthony D Saleh M Pressure Ulcer in Jordan: A point prevalence Journal of tissue viability 2011 20 14 19 10.1016/j.jtv.2010.08.001 20880710

[B20] Ministry of Health Annual Statistical Book 2009 Amman, Jordan: Ministry of Health http://www.moh.gov.jo/EN/Pages/Periodic-Newsletters.aspx

[B21] Vanderwee K Clark M Dealey C Gunningberg L Defloor T Pressure ulcer prevalence in Europe: a pilot study J Eval Clin Pract 2007 13 2 227 235 10.1111/j.1365-2753.2006.00684.x 17378869

[B22] Dealey C Defloor T Prevention Pressure Ulcer Guidelines 2009 Arlington, Virginia (D.C. Area): Presentation at the 11th National Pressure Ulcer Advisory Panel National Biennial Conference February 27, 2009

[B23] Pieper B Mott M Nurses’ knowledge of pressure ulcer prevention, staging, and description Adv Wound Care 1995 8 3 34 38, 40 7795877

[B24] Abou El Enein NY Zaghloul AA Nurses’ knowledge of prevention and management of pressure ulcer at a Health Insurance Hospital in Alexandria Int J Nur Prac 2011 17 3 262 268 10.1111/j.1440-172X.2011.01933.x 21605266

[B25] Hayajneh YA AbuAlRub RF Athamneh AZ Almakhzoomy IK Turnover rate among registered nurses in Jordanian hospitals: an exploratory study Int J Nur Prac 2009 15 4 303 310 10.1111/j.1440-172X.2009.01758.x 19703047

[B26] Hulsenboom MA Bours GJ Halfens RJ Knowledge of pressure ulcer prevention: a cross-sectional and comparative study among nurses BioMed Central Nurs 2007 6 2 1 11 10.1186/1472-6955-6-2 PMC1821326 17349049

[B27] Choa I Parkb HA Chunge E Exploring practice variation in preventive pressure-ulcer care using data from a clinical data repository Int J Med Inform 2011 80 47 55 10.1016/j.ijmedinf.2010.10.019 21130682

